# Supplementary material for: HIVprotI: an integrated web based platform for prediction and design of HIV proteins inhibitors
Source: J Cheminform. 2018 Mar 9;10:12. doi: 10.1186/s13321-018-0266-y (PMC5845081; doi:10.1186/s13321-018-0266-y)
Supplement: Supplementary file 2 — Additional file 2. Source code of HIVProtI web server. [file 13321_2018_266_MOESM2_ESM.zip › HIVprotI_Source-code/hivprotI/weka-3-6-6/doc/weka/gui/package-tree.html]

weka.gui Class Hierarchy


---


|  |  |  |
| --- | --- | --- |
| **Overview** \| **Package** \| Class \| **Tree** \| **Deprecated** \| **Index** \| **Help** \| **Weka's home** | |  |
| **PREV**   **NEXT** | **FRAMES**    **NO FRAMES**     **All Classes** |  |


---


## Hierarchy For Package weka.gui

**Package Hierarchies:**: All Packages

---

## Class Hierarchy

- java.lang.Object
  - javax.swing.AbstractListModel (implements javax.swing.ListModel, java.io.Serializable)
    - javax.swing.DefaultListModel
      - weka.gui.**CheckBoxList.CheckBoxListModel**
    - javax.swing.table.AbstractTableModel (implements java.io.Serializable, javax.swing.table.TableModel)
      - weka.gui.**SortedTableModel** (implements javax.swing.event.TableModelListener)
      - weka.gui.**BrowserHelper**- java.awt.Component (implements java.awt.image.ImageObserver, java.awt.MenuContainer, java.io.Serializable)
          - java.awt.Container
            - javax.swing.JComponent (implements java.io.Serializable)
              - javax.swing.AbstractButton (implements java.awt.ItemSelectable, javax.swing.SwingConstants)
                - javax.swing.JToggleButton (implements javax.accessibility.Accessible)
                  - javax.swing.JCheckBox (implements javax.accessibility.Accessible)
                    - weka.gui.**CheckBoxList.CheckBoxListRenderer** (implements javax.swing.ListCellRenderer)
                - javax.swing.JFileChooser (implements javax.accessibility.Accessible)
                  - weka.gui.**ConverterFileChooser**
                  - javax.swing.JInternalFrame (implements javax.accessibility.Accessible, javax.swing.RootPaneContainer, javax.swing.WindowConstants)
                    - weka.gui.**Main.ChildFrameMDI**
                    - javax.swing.JLayeredPane (implements javax.accessibility.Accessible)
                      - javax.swing.JDesktopPane (implements javax.accessibility.Accessible)
                        - weka.gui.**Main.BackgroundDesktopPane**
                      - javax.swing.JList (implements javax.accessibility.Accessible, javax.swing.Scrollable)
                        - weka.gui.**CheckBoxList**
                        - javax.swing.JPanel (implements javax.accessibility.Accessible)
                          - weka.gui.**AttributeListPanel**- weka.gui.**AttributeSelectionPanel**- weka.gui.**AttributeSummaryPanel**- weka.gui.**GenericArrayEditor** (implements java.beans.PropertyEditor)- weka.gui.**GenericObjectEditor.GOEPanel**- weka.gui.**InstancesSummaryPanel**- weka.gui.**LogPanel** (implements weka.gui.Logger, weka.gui.TaskLogger)- weka.gui.**MemoryUsagePanel**- weka.gui.visualize.**PrintablePanel** (implements weka.gui.visualize.PrintableHandler)
                                            - weka.gui.**AttributeVisualizationPanel**
                                            - weka.gui.**PropertyPanel**- weka.gui.**PropertySheetPanel** (implements java.beans.PropertyChangeListener)- weka.gui.**ResultHistoryPanel**- weka.gui.**SetInstancesPanel**- weka.gui.**SimpleCLIPanel** (implements java.awt.event.ActionListener)- weka.gui.**WekaTaskMonitor** (implements weka.gui.TaskLogger)
                          - javax.swing.JPopupMenu (implements javax.accessibility.Accessible, javax.swing.MenuElement)
                            - weka.gui.**GenericObjectEditor.JTreePopupMenu**
              - java.awt.Window (implements javax.accessibility.Accessible)
                - java.awt.Dialog
                  - javax.swing.JDialog (implements javax.accessibility.Accessible, javax.swing.RootPaneContainer, javax.swing.WindowConstants)
                    - weka.gui.**DatabaseConnectionDialog**- weka.gui.**GenericObjectEditor.CapabilitiesFilterDialog**- weka.gui.**ListSelectorDialog**- weka.gui.**PropertyDialog**- weka.gui.**PropertySelectorDialog**- weka.gui.**ViewerDialog** (implements javax.swing.event.ChangeListener)
                  - java.awt.Frame (implements java.awt.MenuContainer)
                    - javax.swing.JFrame (implements javax.accessibility.Accessible, javax.swing.RootPaneContainer, javax.swing.WindowConstants)
                      - weka.gui.**GUIChooser**- weka.gui.**GUIChooser.ChildFrameSDI**- weka.gui.**LogWindow** (implements javax.swing.event.CaretListener, javax.swing.event.ChangeListener)- weka.gui.**Main** (implements weka.core.OptionHandler)- weka.gui.**Main.ChildFrameSDI**
                      - weka.gui.**SimpleCLI**
                    - weka.gui.**SplashWindow**
          - weka.gui.**ComponentHelper**- weka.gui.**CostMatrixEditor** (implements java.beans.PropertyEditor)- javax.swing.tree.DefaultMutableTreeNode (implements java.lang.Cloneable, javax.swing.tree.MutableTreeNode, java.io.Serializable)
                - weka.gui.**GenericObjectEditor.GOETreeNode**
                - javax.swing.filechooser.FileFilter
                  - weka.gui.**ExtensionFileFilter** (implements java.io.FilenameFilter, java.io.Serializable)
                  - weka.gui.**GenericObjectEditor** (implements weka.gui.CustomPanelSupplier, java.beans.PropertyEditor)- weka.gui.**GenericPropertiesCreator**- weka.gui.**HierarchyPropertyParser** (implements java.io.Serializable)- weka.gui.**JListHelper**- weka.gui.**JTableHelper**- java.awt.event.KeyAdapter (implements java.awt.event.KeyListener)
                              - weka.gui.**ResultHistoryPanel.RKeyAdapter** (implements java.io.Serializable)
                              - weka.gui.**Loader**- weka.gui.**LookAndFeel**- weka.gui.**Messages**- java.awt.event.MouseAdapter (implements java.awt.event.MouseListener, java.awt.event.MouseMotionListener, java.awt.event.MouseWheelListener)
                                      - weka.gui.**ResultHistoryPanel.RMouseAdapter** (implements java.io.Serializable)
                                      - java.beans.PropertyEditorSupport (implements java.beans.PropertyEditor)
                                        - weka.gui.**FileEditor**- weka.gui.**SelectedTagEditor**
                                        - weka.gui.**SaveBuffer**- weka.gui.**SimpleCLIPanel.CommandlineCompletion**- weka.gui.**SimpleDateFormatEditor** (implements java.beans.PropertyEditor)- weka.gui.**SortedTableModel.SortContainer** (implements java.lang.Comparable<T>)- weka.gui.**SysErrLog** (implements weka.gui.Logger)

## Interface Hierarchy

- weka.gui.**CustomPanelSupplier**- weka.gui.**Logger**- weka.gui.**MainMenuExtension**- weka.gui.**TaskLogger**

---


|  |  |  |
| --- | --- | --- |
| **Overview** \| **Package** \| Class \| **Tree** \| **Deprecated** \| **Index** \| **Help** \| **Weka's home** | |  |
| **PREV**   **NEXT** | **FRAMES**    **NO FRAMES**     **All Classes** |  |


---
